# The Impact of Heavy Menstrual Bleeding on Quality of Life: A Mixed‐Methods Survey Study

**DOI:** 10.1111/1471-0528.70290

**Published:** 2026-06-21

**Authors:** Michalina Drejza, Lily S. Nicholson, Gabrielle Paquette, Mehar Chawla, Claire L. Vale, Ewelina Rogozińska, Ngawai Moss, Niccola Hutchinson‐Pascal, Bassel H. Al Wattar

**Affiliations:** ^1^ Cambridge University Hospitals NHS Foundation Trust Cambridge UK; ^2^ UCL Innovative Clinical Trials Unit UCL London UK; ^3^ EGA Institute for Women's Health UCL London UK; ^4^ Imperial College Healthcare NHS Trust London UK; ^5^ Patient and Public Contributor, Katie's Team London UK; ^6^ Co‐Production Collective University College London London UK; ^7^ Clinical Trials Unit, Faculty of Health Medicine and Social Care Anglia Ruskin University Chelmsford UK; ^8^ Beginnings Assisted Conception Unit Epsom and St Helier University Hospitals London UK

**Keywords:** heavy menstrual bleeding, menstrual health, mixed‐methods, psychosocial impact, quality of life

## Abstract

**Objective:**

To examine the physical, psychological, social, and functional impact of heavy menstrual bleeding (HMB) using a mixed‐methods approach.

**Design:**

Mixed‐methods online survey.

**Population:**

A UK‐wide sample of adults assigned female at birth with lived experience of HMB. Participants were recruited between June and July 2024 via social media, menstrual‐health charities and the Katie's Team and Co‐Production Collective networks.

**Methods:**

Participants completed an online questionnaire comprising multiple‐choice and open‐ended items. Qualitative data were analysed inductively using thematic analysis and quantitative data were summarised descriptively. Findings were integrated to understand the multi‐dimensional impact of HMB. Patient and public involvement informed survey development.

**Main Outcome Measures:**

Menstrual cycle characteristics; self‐reported physical, psychological, social, and functional impacts of HMB.

**Results:**

105 participants were included. Most reported bleeding lasting ≥ 6 days and long‐standing HMB beginning early in their reproductive lives. Severe or very severe impact was reported across physical health (83%), mental health (73%), social participation (83%), and sexual relationships (59%). Qualitative findings highlighted profound fatigue, symptoms consistent with anaemia, pain, cognitive difficulties, and night‐time disruption. Participants described pervasive anxiety regarding leakage, altered career or study trajectories, and adverse effects on intimacy and relationships. Many structured daily routines around access to toilets and sanitary protection. Experiences of stigma and unmet healthcare needs were common.

**Conclusions:**

Among participants, HMB was associated with quality of life impairment across multiple domains, extending far beyond bleeding volume. These findings underscore the importance of holistic, patient‐centred management, including timely investigation, appropriate medical therapy, and psychosocial support.

## 1 Introduction

Heavy menstrual bleeding (HMB) is one of the most common gynaecological complaints, affecting approximately one in three women and people who menstruate of reproductive age worldwide [1, 2]. Prevalence estimates vary by population, with large epidemiological surveys reporting rates between 18% and 38% [3, 4]. The National Institute for Health and Care Excellence (NICE) defines HMB as excessive menstrual blood loss that interferes with a woman's physical, emotional, social, and material quality of life [5]. This represents a shift away from earlier volume‐based thresholds (e.g., > 80 mL per cycle) towards a patient‐centred framework prioritising disruption to daily life.

The most widely recognised physical complication of HMB is iron‐deficiency anaemia and its associated symptoms, with severe cases occasionally requiring emergency care or surgical intervention [6]. Even in the absence of anaemia, sustained HMB and iron‐deficiency can significantly impair energy levels, well‐being and overall quality of life [7, 8]. Quantitative studies consistently report lower mental health scores on validated quality of life instruments among women with HMB compared with controls [9, 10], while qualitative research highlights persistent anxiety about leakage and feelings of shame [11, 12]. These physical and psychological effects translate into substantial functional impairment, disrupting participation in education, employment, and social life. HMB is a leading cause of school absenteeism and reduced participation in extracurricular activities among adolescents [13], and among working‐age adults it is associated with increased sick leave, reduced productivity, and career modification [14, 15], with an estimated annual cost of £11 billion to the UK economy [16]. Beyond work and study, women with HMB frequently avoid activities because of fear of leakage or inadequate facilities, contributing to social withdrawal and isolation, particularly in contexts where menstrual stigma persists [11]. Intimate and sexual relationships are also adversely affected, as pain, fatigue, and concern about bleeding during intercourse reduce sexual desire and frequency [11].

While quantitative studies have clarified the prevalence and measurable outcomes of HMB, they have not fully captured the lived experience of affected individuals. A mixed‐methods approach that integrates qualitative methods to provide insights into coping strategies, perceived stigma, and disruptions to daily routines, integrated with numerical trends, can produce findings that are both statistically robust and clinically meaningful. Therefore, we aimed to conduct a mixed‐methods study to assess the menstrual cycle characteristics in women with HMB and the impact of HMB on quality of life.

## 2 Methods

### 2.1 Study Population and Recruitment

Eligible participants included individuals assigned female at birth, aged 18 or older, who had lived experience of HMB and were based in the UK. Participants were recruited via social media and partner charities including Menstrual Health Project between June and July 2024. The social media advert used to recruit participants is available in Figure S1. Informed consent was obtained from each participant prior to completing the survey (Appendix S1).

### 2.2 Survey Development

An online survey was developed to examine the impact of HMB on quality of life, as well as participant demographics and menstrual cycle characteristics. Feedback on the survey design was obtained from two online patient and public involvement (PPI) workshops of 10 women with lived experience of HMB facilitated by NM and NHP. Participants helped refine the wording of questions; for example, the term ‘intimacy’ was replaced by ‘relationships and sex life’. Participants also highlighted areas that are not routinely captured in clinical questionnaires including financial consequences of HMB, neurodiversity, and gender, which were incorporated into the final survey design (Appendix S2).

### 2.3 Study Design

This study utilised a convergent design, where quantitative and qualitative data were collected and analysed in parallel and integrated at interpretation [17]. Multiple‐choice questions were used to assess participant demographics, menstrual cycle characteristics, and the magnitude of the impact of HMB on quality of life. Participants rated the impact of HMB across quality of life domains (physical health, mental health, work life or studies, social activities, relationships and sex life) on a scale from 1 to 5. Open‐ended questions explored how HMB impacts across each domain and other aspects of life which were not otherwise captured.

The questionnaire also included questions examining participant experiences seeking treatment for HMB and their treatment preferences. These data will be reported separately.

### 2.4 Data Analysis

All survey responses were included in data analyses regardless of survey completion status.

Participant demographics, menstrual cycle characteristics and quantitative data assessing the impact of HMB on quality of life were analysed descriptively and presented as frequencies and percentages. When analysing the cause of HMB, participants were able to select multiple causes and contribute to more than one diagnostic category. For example, if a participant reported ‘Adenomyosis and Endometriosis’, that participant was included in both the Adenomyosis and Endometriosis categories.

Open‐ended responses were analysed using reflexive thematic analysis in NVivo 12 [18, 19]. After familiarisation with the data, MD and GP independently coded responses using an inductive approach. Investigators had clinical and research experience relevant to women's health and qualitative research and reflexive practice was incorporated throughout the analytic process to acknowledge how prior disciplinary and clinical perspectives could shape interpretation of participant accounts. Coding was undertaken independently, with discrepancies resolved through discussion to ensure that potential themes were not overlooked. Initial codes were subsequently grouped into broader themes.

The qualitative component was the dominant method providing in‐depth insights into the impact of HMB on quality of life, with quantitative data providing complementary context. Qualitative and quantitative data were integrated narratively at the reporting stage to identify areas of convergence or divergence. Integration was undertaken by MD, with discussion and input from LN and GP. This study was reported using the Good Reporting of A Mixed Methods Study (GRAMMS) checklist (Table S1) [20].

## 3 Results

### 3.1 Population Characteristics

One hundred and seven participants completed the survey; however, responses from two participants who stated they did not have a lived experience of HMB were excluded. Therefore, responses from 105 participants were included in the study. Self‐described participant characteristics (Table 1) were distributed across reproductive ages, with the largest age groups being 25–34 years (35/105, 32.7%) and 35–44 years (35/105, 32.7%). Participants were located across the UK, with the largest single‐region contribution from Greater London (29/105, 27.1%), followed by Wales (17/105, 15.9%). The sample was majority White (81/105, 75.7%), with Asian or Asian British (9/105, 8.4%), Black or Black British (7/105, 6.5%) and mixed/multiple ethnic groups represented in smaller numbers. Educational attainment was high, with 72% (*n* = 77) having an undergraduate or postgraduate qualification and the majority of participants (58/105, 54.2%) stated that they were employed full‐time. Overall, 21.5% (*n* = 23) identified as having a disability and 29.0% (*n* = 31) identified as neurodivergent.

**TABLE 1:** Self‐described population characteristics (*n* = 105).

Variable	Characteristics	*N* (%)
Age	18–24	13 (12.1)
	25–34	35 (32.7)
	35–44	35 (32.7)
	45+	19 (17.8)
	Unknown	3 (2.8)
UK region	Greater London	29 (27.1)
	Wales	17 (15.9)
	South East	11 (10.2)
	East of England	10 (9.3)
	North West	9 (8.4)
	South West	6 (5.6)
	West Midlands	5 (4.7)
	Yorkshire and Humber	5 (4.7)
	East Midlands	3 (2.8)
	North East	3 (2.8)
	Northern Ireland	2 (1.9)
	Scotland	2 (1.9)
	Unknown	3 (2.8)
Ethnicity	White	81 (75.7)
	Asian or Asian British	9 (8.4)
	Black, Black British, Caribbean or African	7 (6.5)
	Mixed or multiple ethnic groups	3 (2.8)
	Unknown	5 (4.8)
Education	Postgraduate degree or equivalent	43 (40.2)
	Undergraduate degree or equivalent	34 (31.8)
	A‐levels or equivalent	18 (16.8)
	GCSEs	6 (5.6)
	Unknown	4 (3.8)
Employment	Employed; full‐time	58 (54.2)
	Employed; part‐time	16 (15.0)
	Student	10 (9.3)
	Self employed	7 (6.5)
	Unemployed	3 (2.8)
	Other	7 (6.7)
	Unknown	3 (2.8)
Disability	No	78 (72.9)
	Yes	23 (21.5)
	Unknown	4 (3.8)
Neurodivergence	No	68 (63.5)
	Yes	31 (29.0)
	Unknown	6 (5.7)
Gender identity	Female	100 (93.4)
	Non‐binary	2 (1.9)
	Unknown	3 (2.8)

### 3.2 Characteristics of Menstrual Cycle

For menstrual cycle characteristics (Table 2), the duration of menstrual bleeding was most commonly reported as 6–7 days (45/105, 42.9%), followed by 8 days or more (30/105, 29.0%). Menstrual cycle regularity was varied, 22/105 (21.0%) participants indicated their cycles were always regular, 19/105 (18.1%) reported never regular, with others reporting their cycles were often or sometimes regular. The majority of respondents (72/105, 68.6%) reported cycle lengths between 21 and 35 days, while others reported shorter or longer cycle lengths.

**TABLE 2:** Menstrual cycle characteristics (*n* = 105).

Variable	Characteristics	*N* (%)
How many days do you bleed?	1–3	1 (1.0)
	4–5	25 (23.8)
	6–7	45 (42.9)
	8+	30 (29.0)
	Variable	1 (1.0)
	Unknown	3 (2.9)
How regular is your menstrual cycle?	Often	33 (31.4)
	Sometimes	28 (26.7)
	Always	22 (21.0)
	Never	19 (18.1)
	Unknown	3 (2.9)
How long is your menstrual cycle?	20 days or less	12 (11.4)
	21–35 days	72 (68.6)
	36+ days	12 (11.4)
	Varies	4 (3.8)
	Unknown	5 (4.8)
When did you begin experiencing HMB?	Always heavy	79 (75.2)
	After giving birth	7 (6.7)
	Perimenopause	6 (5.7)
	After no particular event, but have not always had them	1 (1.0)
	After stopping the contraceptive pill	1 (1.0)
	Other	5 (4.8)
	Unknown	6 (5.7)

The vast majority of participants (79/105, 75.2%) reported consistently experiencing HMB throughout their reproductive lives (Table 2). A smaller subset of respondents identified HMB as emerging in connection with reproductive or hormonal changes, such as following childbirth (7/105, 6.7%), or when approaching menopause (6/105, 5.7%).

### 3.3 Causes of Heavy Menstrual Bleeding

Participants most commonly stated there was ‘no known cause’ (47/105, 44.8%) linked to their HMB, followed by endometriosis (29/105, 27.6%); adenomyosis (15/105, 14.3%), fibroids (11/105, 10.5%), PCOS (7/105, 6.7%) or combinations of pathologies (e.g., adenomyosis with endometriosis or fibroids) (Table 3). A small number of participants reported rarer conditions linked with HMB (e.g., von Willebrand disease).

**TABLE 3:** Self‐reported causes of heavy menstrual bleeding (*n* = 105).

Condition	*N* (%)
No known cause	47 (44.8)
Endometriosis	29 (27.6)
Adenomyosis	15 (14.3)
Fibroids	11 (10.5)
PCOS	7 (6.7)
Polyps	2 (1.9)
Cysts	2 (1.9)
Endosalpingiosis	1 (1.0)
Copper Coil made HMB worse	1 (1.0)
Under investigation	1 (1.0)
Uterine cervical stenosis	1 (1.0)
Von Willebrand disease	1 (1.0)
Covid Jab (related)	1 (1.0)
Unknown	3 (2.9)

### 3.4 Influence on Different Aspects of Health

#### 3.4.1 Physical Health

The majority of participants reported very severe (score 5) consequences of HMB on their physical health (65/105, 61.9%). An additional 22/105 (21.0%) participants reported severe impact (score 4), indicating that over 80% of respondents experienced high‐level physical impairment due to HMB (Figure 1a).

**FIGURE 1:**
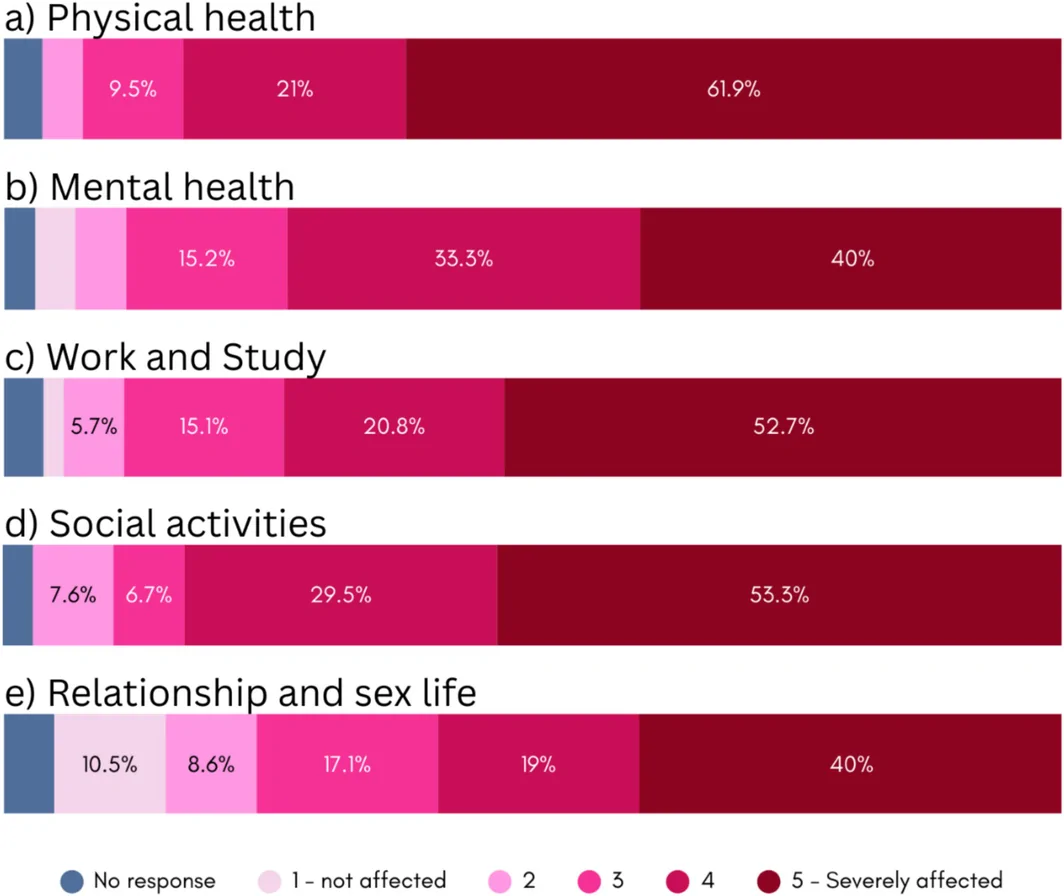
Self‐reported impact of heavy menstrual bleeding on quality of life domains on a scale from 1 to 5, with 1 being no impact and 5 being very severe impact (*n* = 105). (a) physical health, (b) mental health, (c) work and study, (d) social activities, and (e) relationships and sex life.

In the open‐ended responses, participants consistently described symptoms of anaemia as one of the most debilitating consequences of HMB, with multiple participants linking their symptoms directly to blood loss. Experiences ranged from ‘feeling faint and dizzy especially during my periods’ (P44) or ‘exhausted and weak…with headaches and nausea’ (P5), to more severe cases where hospitalisation and blood transfusions were required: ‘I nearly died when I passed out without warning and woke up two days later in hospital after two emergency blood transfusions’ (P83). Alongside systemic effects, participants reported significant pain, often described as ‘*excruciating*’ (P17), ‘*debilitating*’ (P47), or leaving them ‘unable to go about normal living’ (P54). For some, pain was the most distressing symptom, outweighing bleeding itself: ‘The thing that bothers me the most about my period is the pain and nausea, more so than the heavy bleeding itself’ (P18). Other physical and cognitive impacts included insomnia, with participants reporting frequent night waking due to pain or urinary urgency, as well as bloating and brain fog, which compounded feelings of physical depletion.

#### 3.4.2 Mental Health

The effect of HMB on mental health was substantial, with the majority of participants reporting either severe (35/105, 33.3%) or very severe (42/105, 40.0%) impact (Figure 1b). A prominent theme that emerged was anxiety related to the unpredictability, intensity, and social consequences of HMB. Participants described constant fear of leaking through clothing, bedding or furniture and the resulting need to plan their daily lives around access to toilets and sanitary products. As one participant stated, ‘It has been hell… I bleed through everything, often coming through my clothes. This causes extreme embarrassment and paranoia’ (P90). The psychological toll was evident in reports of avoidance behaviours: ‘I get anxious about going out, especially in summer when clothes are lighter and thinner’ (P5), and ‘Social anxiety about leaking outside so I tend to stay in at least for the first half of the week or so that I am bleeding’ (P74). Several participants described diagnosed mental health conditions, with one reporting, ‘I have been diagnosed with PTSD due to my heavy periods, hemorrhages and endometriosis. This followed with crippling anxiety’ (P79).

In addition to anxiety, many participants highlighted low mood and mood swings associated with HMB. For example, one participant said, ‘Always have [a] very low mood 1 week before my period’ (P11). At the same time, another described, ‘The week before my period my mood changes incredibly, I am incredibly irritable and cry for no reason. I get quite depressed even though I am on antidepressants’ (P13). Some described a more chronic cycle of instability: ‘I have mood swings throughout my cycle, both pre‐menstrual and during periods. This adds up to 3/4 weeks of each month!’ (P58).

Feelings of sadness and distress also featured, particularly around the humiliation and loss of control associated with HMB. Participants reflected on their experiences from adolescence, describing menstruation as ‘hell to live with’ (P99) and ‘so humiliating and draining’ (P45). For some, the heavy bleeding combined with pain and medication use left them feeling overwhelmed: ‘…I am on so much medication’ (P71).

#### 3.4.3 School, Work and Social Activities

Work and study activities were also significantly disrupted, with 56/105 (52.7%) reporting very severe impact and 22/105 (20.8%) reporting work and study activities were severely impacted (Figure 1c). Social activities were among the most profoundly affected domains, with 56/105 (53.3%) reporting very severe impact on social activities, and none of the participants reported no impact (Figure 1d).

Many participants reported difficulty concentrating due to pain, fatigue, or fear of leaking. For instance, ‘Finding it hard to function at work’ (P29) and ‘Harder to concentrate whilst in pain or needing to change frequently’ (P7) were common sentiments. One participant explained, ‘I usually feel quite ill for at least one day every month… it's especially a problem if I have lots of meetings and I need to be in the office on this day, as it can feel like there's nowhere to hide’ (P18).

The need to take sick leave was repeatedly described, with participants missing work, university, or social activities. Some reported having to reduce their hours or change jobs entirely: ‘I have to cut down my working hours, I am not a normal [20‐30] year old’ (P71), while another shared, ‘I deliberately sought a working from home job… as that way on the 1–2 days each month when I am at my worst physically, I can still work’ (P33). More extreme accounts revealed that education and career paths had been derailed: ‘I had to defer my degree as my periods were stopping me from studying. I no longer work and am on the full amount of [Personal Independence Payment] PIP and [Employment and Support Allowance] ESA benefits’ (P65).

Social isolation and lost opportunities were commonly reported, such as: ‘[I] usually end up having to cancel plans as I'm in pain and worried about bleeding through clothes’ (P55). Participants also mentioned the burden of day‐to‐day planning and coping strategies needed to manage their symptoms, with one participant stating, ‘You have to plan your day around being close to the toilet as my flow is so heavy I have to go… every hour’ (P66).

#### 3.4.4 Relationships and Sex Life

Sexual activity was significantly affected, with 59% (62/105) of respondents describing severe or very severe impairment in their relationships and sex life (Figure 1e). Many reported that sex became physically painful, emotionally distressing, or altogether impossible during large portions of the month. For some, the experience of heavy bleeding, clotting, and associated pelvic pain created an immediate barrier to sexual activity: ‘Since they have become this heavy, sex is almost impossible’ (P14); ‘In my recent relationship, we pretty much didn't have sex for months at a time. He didn't understand how painful sex was for me and I was fed up [with] explaining’ (P51).

The physical and emotional toll of HMB shaped participants' sense of sexual self‐worth and desirability. Many expressed that they did not feel attractive, desirable or sexy due to the combination of bloating, fatigue, pelvic pain, and ongoing bleeding: ‘I don't feel desirable because I feel so unwell for half of the month’ (P8); ‘I feel very unsexy most of the time due to bloat and chronic pelvic pain’ (P75). The unpredictability of bleeding episodes also created anxiety around intimacy and contributed to fears of rejection. One participant described being ‘scared of being intimate’ (P40), while another reflected that her symptoms had ‘destroyed most chances [she'd] had at relationships so far’ (P84). Several participants recalled highly distressing experiences, such as staining bedsheets during sexual activity, which they described as ‘*mortifying*’ (P13, P86), further reinforcing feelings of shame and avoidance.

Relational strain was a recurrent theme. Single participants frequently emphasised the difficulties of navigating new relationships while managing unpredictable bleeding and pain: ‘Being single, it's hard to know how to navigate it’ (P46). Participants in relationships also described tension arising from HMB: ‘Period pain stops me from having sex with my partner, which causes tension in my relationship’ (P36). Some reported that partners withdrew or ended relationships because of the perceived burden of care: ‘can lead to break up as he didn't want to care for me and it's too much work for them’ (P15). Others highlighted how HMB altered the dynamics of their relationships, shifting them into a carer–patient role: ‘My partner has to care for me when I am on my period’ (P17); ‘Pain after sex, husband having to care for me. It changes your relationship’ (P91). However, a small number of participants reflected on the buffering role of partner support. For example, one woman explained that although she avoided sex during heavy flow days, her ‘understanding and supportive partner’ meant this did not strain their relationship (P18).

## 4 Discussion

### 4.1 Main Findings

This mixed‐methods survey of 105 participants examined how HMB affects quality of life across physical, psychological, social and functional domains. Participants described substantial impacts including fatigue and anaemia‐like symptoms, pain, cognitive complaints like ‘brain fog’, anxiety and low mood, alongside disruptions to work, education and social life. Participants also reported reduced sexual activity due to embarrassment or pain, lowered self‐esteem, and in some cases, relationship breakdown. This mixed‐methods data highlights the broad and multifaceted burden of HMB on daily functioning and wellbeing among participants.

### 4.2 Strengths and Limitations

Key strengths of the study include its mixed‐methods design, which allowed numerical description of participant characteristics to be integrated with rich qualitative accounts capturing the lived meaning of symptoms and their downstream effects. The UK‐wide recruitment and broad age range support transferability of findings to similar health‐service contexts, while the qualitative data provided detailed insights into how HMB impacts quality of life.

However, important limitations temper interpretation. The convenient, self‐selected nature of recruitment is likely to have skewed the sample towards people with more severe or persistent symptoms and those highly motivated to participate, introducing significant selection bias and limiting generalisability. Demographics skewed towards White and highly educated participants, restricting insights into the experiences of ethnic minority groups, people with lower levels of education and gender‐diverse people. Given the small, self‐selected sample, inferential and subgroup analyses were not undertaken to avoid overinterpretation. In addition, collecting qualitative data using survey methods limited opportunities to probe participants for more detail compared to using interview methods.

### 4.3 Interpretation

Our findings illustrate that, for participants in this study, HMB was experienced as a condition with profound and often under‐recognised consequences on multiple aspects of quality of life. Similar impacts on daily functioning and wellbeing have been described in previous qualitative studies [11, 12]. Reports of fatigue, ‘brain fog’, anxiety, and anticipatory stress related to leakage mirror symptoms in other work on HMB and iron deficiency, including cognitive and emotional impacts [7, 9, 10].

These findings highlight limitations of traditional HMB assessment approaches that focus on bleeding volume alone, and may not capture the functional, emotional and social consequences described by participants [21, 22]. Our qualitative findings suggest a substantial ‘hidden burden’ of HMB, characterised by heightened vigilance, modified daily routines, avoidance behaviours and diminished self‐esteem, which may not be apparent in routine clinical encounters unless actively explored.

Taken together, these findings suggest that HMB was commonly experienced as a condition with systemic and psychosocial consequences, rather than a purely gynaecological symptom. Focusing only on biomedical factors may risk underestimating severity, delaying appropriate intervention and perpetuating feelings of dismissal or stigma reported by participants in this study and others [22, 23].

### 4.4 Implications for Clinical Practice

These findings have important implications for clinical care. Clinicians should acknowledge and validate the hidden burden of HMB and routinely assess the impact of HMB on quality of life alongside menstrual characteristics. Participants frequently described long‐standing symptoms that were poorly recognised or insufficiently addressed, particularly when no clear underlying cause was identified.

Clinician enquiry should routinely extend beyond bleeding duration and volume to include fatigue, cognitive function, mental health, social participation, work or educational functioning, effects on relationships and sexual wellbeing and practical and financial implications of HMB. Normalising these discussions may help reduce stigma and encourage disclosure of concerns that patients might otherwise minimise or withhold.

Patients should be supported to evaluate how HMB affects different aspects of their lives. Supporting patients to articulate what matters most to them, whether symptom control, energy levels, work participation or intimacy, can guide more individualised management strategies. This approach aligns with patient‐centred care and may improve satisfaction and treatment adherence.

### 4.5 Implications for Future Research

Future research should prioritise the development and validation of reliable, patient‐centred measurement instruments to assess the quality of life impact of HMB. Existing measures may not capture the cumulative, cyclical, and anticipatory nature of impairment described by participants. Measurement instruments should be feasible for both research and clinical settings, sensitive to change over time, and inclusive of diverse populations.

In addition, future studies should evaluate the feasibility and effectiveness of incorporating routine quality of life assessment into standard HMB care pathways. This may help clarify effects on clinical decision‐making, patient outcomes and healthcare utilisation. Future studies should also explore the practical and financial burden of HMB, and associated coping strategies. Population‐representative studies are also needed to capture experiences across socioeconomic, ethnic, and gender‐diverse groups, who remain under‐represented in much of the current literature.

## 5 Conclusion

Among participants in this study, HMB was associated with reduced quality of life across physical, psychological, social, and functional domains. Reasonable inferences from these findings support the importance of routine assessment of HMB and its quality of life impact within primary care and gynaecology consultations, including evaluation of fatigue, pain, mental wellbeing, work and social functioning, and symptoms of iron deficiency and anaemia. Earlier recognition and investigation may help reduce delays in referral and treatment. Improved access to evidence‐based therapies, alongside clearer referral pathways, may further improve outcomes for patients whose symptoms significantly impair daily functioning or do not respond to first‐line management. Greater awareness of HMB as a significant health issue, enhanced clinician education, and incorporation of patient‐reported outcome measures into routine care may also help address its broader psychosocial impact.

## Author Contributions

M.D. conducted analyses and drafted the final manuscript. L.S.N. contributed to the survey design, data analysis, and drafted the final manuscript. G.P. and M.C. contributed to the survey design and data analysis. C.L.V. served as the project co‐chief investigator and contributed to survey design. E.R. provided input to the initial study design. N.M. and N.H.‐P. organised and conducted the P.P.I. workshops which informed the survey development. B.H.A.W. conceived the idea and served as project co‐chief investigator. All authors provided critical input to the final manuscript.

## Funding

This study was funded by the NIHR HTA Evidence Synthesis Programme (NIHR153187).

## Ethics Statement

Approval for this study was obtained from the Institute for Women's Health Low Risk Ethics Committee at University College London (IfWH_LREC_004_2023_24).

## Conflicts of Interest

The authors declare no conflicts of interest.

## Supporting information


**Appendix S1:** Participant information and consent form.

**Appendix S2:** Heavy menstrual bleeding questionnaire.

**Figure S1:** Social media advert for recruiting participants.

**Table S1:** Good Reporting of A Mixed Methods Study (GRAMMS) checklist.

## Data Availability

Research data are not shared.
