# Supplementary material for: The Impact of Heavy Menstrual Bleeding on Quality of Life: A Mixed‐Methods Survey Study
Source: BJOG. 2026 Jun 21;133(12):2170–7. doi: 10.1111/1471-0528.70290 (PMC13659367; doi:10.1111/1471-0528.70290)
Supplement: Supplementary file 1 [file BJO-133-2170-s001.docx]

## Table S1: Good Reporting of A Mixed Methods Study (GRAMMS) checklist

| **Item** | **Reported** |
| --- | --- |
| Describe the justification for using a mixed methods approach to the research question | Introduction |
| Describe each method in terms of purpose, priority and sequence of methods | Purpose and Sequence of methods – Methods (Study Design)  Priority – Data Analysis |
| Describe each method in terms of sampling, data collection and analysis | Sampling and Data Collection – Methods (Study Population and Recruitment)  Analysis – Methods (Data Analysis) |
| Describe where integration has occurred, how it has occurred and who has participated in it | Methods (Data Analysis) |
| Describe any limitation of one method associated with the present of the other method | Discussion (Strengths and Limitations) |
| Describe any insights gained from mixing or integrating methods | Discussion (Strengths and Limitations) |

Reference: O’Cathain A, Murphy E, Nicholl J. The quality of mixed methods studies in health services research. J Health Serv Res Policy. 2008; 13(2): 92-98
