# Supplementary material for: The Impact of Heavy Menstrual Bleeding on Quality of Life: A Mixed‐Methods Survey Study
Source: BJOG. 2026 Jun 21;133(12):2170–7. doi: 10.1111/1471-0528.70290 (PMC13659367; doi:10.1111/1471-0528.70290)
Supplement: Supplementary file 3 [file BJO-133-2170-s003.docx]

## Appendix S1. Participant Information and Consent Form

**Title of Study:** Qualitative study of the impact of heavy menstrual bleeding on women’s quality of life, their access to treatment, and treatment preferences
**Name and Contact Details of the Researcher(s):** Lily Nicholson, lily.nicholson@ucl.ac.uk
**Name and Contact Details of the Principal Researcher:** Dr Bassel Al Wattar, b.wattar@nhs.net
**Name and Contact Details of the UCL Data Protection Officer:** data-protection@ucl.ac.uk
**This study has been approved by the UCL Research Ethics Committee:**
**Project ID number: 26915/001**

Thank you for considering participation in our research study titled: Qualitative study of the impact of heavy menstrual bleeding on women’s quality of life, their access to treatment, and treatment preferences.

Before you agree to participate, it is important that you understand what this research entails. Please take time to read the following information carefully and discuss it with others if you wish.  Ask us if there is anything that is not clear or if you would like more information.  Please take time to decide whether or not you wish to take part. Thank you for your time. 

Participation involves completing an online survey exploring the experience of women suffering with heavy menstrual bleeding. The survey should take 15-20 minutes of your time. We aim to understand the personal impact of your condition, how it affects your day-to-day life, and your experiences of seeking and receiving treatment. The insights gained will be invaluable in enhancing the care for women who face similar challenges with heavy menstrual bleeding.

Your involvement in the study is entirely voluntary, and you may withdraw at any time without any penalty or loss of benefits to which you are otherwise entitled. Should this questionnaire raise any issues or concerns for you, we strongly urge you to contact your GP. All of your personal information will remain confidential and stored anonymously and securely. We request that you refrain from including any details in the survey that could personally identify you or others, such as your name or names of healthcare providers, to maintain the anonymity of your response. You may withdraw your data from this research up to 31 July 2024. 

**Data Protection Privacy Notice**
The UCL Data Protection Officer provides oversight of UCL activities involving the processing of personal data, and can be contacted at data-protection@ucl.ac.uk

This ‘local’ privacy notice sets out the information that applies to this particular study. For further information on how UCL uses participant information can be found here

The information that is required to be provided to participants under data protection legislation (GDPR and DPA 2018).

The lawful basis that will be used to process your personal data is: ‘public task’ and ‘research purposes’ will be the lawful basis for processing special category data.

Please confirm your understanding and consent by ticking each of the following statements

☐ I confirm that I have read and understood the information for the above study. I have had an opportunity to consider the information and what will be expected of me. I have also had the opportunity to ask questions which have been answered to my satisfaction.

☐ I understand that I will be able to withdraw my data up to 31 July 2024

☐ I understand that my participation is voluntary and that I am free to withdraw at any time without giving a reason. I understand that if I decide to withdraw, any personal data I have provided up to that point will be deleted unless I agree otherwise.

☐ I understand that the data will not be made available to any commercial organisations but is solely the responsibility of the researcher(s) undertaking this study.

☐ I understand that I will not benefit financially from this study or from any possible outcome it may result in in the future.

☐ I understand that the information I have submitted will be published as a report.

☐ I am aware of who I should contact if I wish to lodge a complaint.

☐ I voluntarily agree to take part in this study.
