# Supplementary material for: The Impact of Heavy Menstrual Bleeding on Quality of Life: A Mixed‐Methods Survey Study
Source: BJOG. 2026 Jun 21;133(12):2170–7. doi: 10.1111/1471-0528.70290 (PMC13659367; doi:10.1111/1471-0528.70290)
Supplement: Supplementary file 4 [file BJO-133-2170-s004.docx]

## Appendix S2. Heavy Menstrual Bleeding Questionnaire

Thank you for participating in our survey. Your insights are valuable and will contribute to a better understanding of the impact of heavy menstrual bleeding on women's lives. 

The survey is designed into three sections. In the first section, we aim to get more of an understanding about your periods, including the heaviness and regularity and to explore the broader impact of heavy periods on your daily life including its effect on your work, relationships and overall health. 
The second section seeks to capture your experiences in seeking medical advice and treatment for your heavy periods. Our goal is to gain a comprehensive understanding of how heavy periods have influenced your life. Through this, we aspire to educate and make changes in women dealing with heavy periods.

Lastly, we will gather basic demographic information, including your age, ethnicity, and employment. 
This information will help ensure that our survey reflects a diverse range of perspectives and experiences. We request that you refrain from including any details in the survey that could personally identify you or others, such as your name or names of healthcare providers, to maintain the anonymity of your response.

Should this questionnaire raise any issues or concerns for you, we strongly urge you to contact your GP. The links below are other resources which can be used if you wish to learn more about heavy periods.
https://www.wellbeingofwomen.org.uk/what-we-do/health-information/?category=periods-menstrual-health
https://www.menstrualhealthproject.org.uk/_files/ugd/571191_6f8f8a8c4a9245e3a6de14e481ce2833.pdf

We kindly ask that you answer the questions to the best of your ability, as your responses are crucial in helping us achieve our objectives. No questions are mandatory to complete. 

Thank you once again for your valuable contribution.

**Characteristics of Heavy Periods**

1. **Have you ever suffered from heavy periods?**

- Yes
- No

2. **On average, how many days does your menstrual period last?**

- 1-3 days
- 4-5 days
- 6-7 days
- 8+ days
- Other: please specify

3. **Are your cycles regular?**

- Always
- Often
- Sometimes
- Never

4. **How long on average is your menstrual cycle?**

- 20 days or less
- 21-35 days
- 36 days or more
- Other: please specify

5. **When did you start to experience heavy menstrual bleeding?**

- I have always had heavy periods
- After giving birth
- When approaching menopause
- Other: please specify

6. **Is your heavy menstrual bleeding linked to a known cause?**

- Fibroids
- Adenomyosis
- Endometriosis
- Uterine Polyps
- No known cause
- Other: please specify

7. **On a scale from 1-5, with 1 being no pain and 5 being very severe pain, how painful are your periods?**

| 1 | 2 | 3 | 4 | 5 |
| --- | --- | --- | --- | --- |

**Impact of Heavy Periods on Quality of Life**

8. **What bothers you most about having heavy periods?**

9. **On a scale from 1-5, with 1 being not affected and 5 being severely affected, how much do you think your heavy periods have affected your physical health (e.g. fatigue, nausea, headache, feeling faint)?**

| 1 | 2 | 3 | 4 | 5 |
| --- | --- | --- | --- | --- |

10. **In what way have your heavy periods affected your physical health (e.g. fatigue, nausea, headache, feeling faint)?**

11. **On a scale from 1-5, with 1 being not affected and 5 being severely affected, how much do you think your heavy periods have affected your mental health (e.g. depression, anxiety, mood swings)?**

| 1 | 2 | 3 | 4 | 5 |
| --- | --- | --- | --- | --- |

12. **In what way have your heavy periods affected your mental health (e.g. depression, anxiety, mood swings)?**

13. **On a scale from 1-5, with 1 being not affected and 5 being severely affected, how much do you think your heavy periods have affected your work life/studies?**

| 1 | 2 | 3 | 4 | 5 |
| --- | --- | --- | --- | --- |

14. **In what way have your heavy periods affected your work life/studies?**

15. **On a scale from 1-5, with 1 being not affected and 5 being severely affected, how much do you think your heavy periods have affected your ability to enjoy social activities?**

| 1 | 2 | 3 | 4 | 5 |
| --- | --- | --- | --- | --- |

16. **In what way have your heavy periods affected your ability to enjoy social activities?**

17. **On a scale from 1-5, with 1 being not affected and 5 being severely affected, how much do you think your heavy periods have affected your relationships and sex life?**

| 1 | 2 | 3 | 4 | 5 |
| --- | --- | --- | --- | --- |

18. **In what way have your heavy periods affected your relationships and sex life?**

19. **Have heavy periods affected your quality of life in any other ways (e.g. cultural practices, happiness, freedoms, finances)?**

**Seeking Treatment and Medical Advice**

20. **Have you ever sought treatment or medical help to manage heavy periods?**

- Yes
- No

**If Yes,**

21. **On a scale from 1-5, with 1 being very difficult and 5 being very easy, how easy did you feel it was to seek medical help and treatment for your heavy periods in the NHS?**

| 1 | 2 | 3 | 4 | 5 |
| --- | --- | --- | --- | --- |

22. **On a scale from 1-5, with 1 being ‘did not feel supported’ and 5 being ‘felt very supported’, how much did you feel heard and supported when accessing treatment for your heavy periods in the NHS?**

| 1 | 2 | 3 | 4 | 5 |
| --- | --- | --- | --- | --- |

23. **On a scale from 1-5, with 1 being not confident at all and 5 being extremely confident, how confident did you feel about your understanding of the treatment options being explained to you?**

| 1 | 2 | 3 | 4 | 5 |
| --- | --- | --- | --- | --- |

24. **Please describe your experience seeking treatment for your heavy periods in the NHS and tell us more about your treatment journey?**

25. **Are you concerned about any long-term impacts of the treatment(s) you have received?**

26. **What positive effects / benefits (if any) did you experience from your treatment(s)?**

27. **What side effects or adverse effects (if any) did you experience from your treatment(s)?**

**If No,**

28. **Please tell us about any reasons why you have not sought treatment for your heavy periods?**

**All respondents,**

29. **When choosing a treatment for heavy periods, what are / would be your priorities? Please consider the following: treatment safety, chance of treatment success, impact on fertility, ease of treatment, availability on the NHS, cost.**

**Demographic information**

30. **Please select you age range:**

- 18-24
- 25-34
- 35-44
- 45+

31. **Which of the following regions best describes where you are currently based?**

- Greater London
- Wales
- Scotland
- Northern Ireland
- West Midlands
- East Midlands
- North East (e.g. Newcastle)
- North West (e.g. Manchester, Liverpool)
- Yorkshire and Humber
- East of England (e.g. Cambridge, Norwich)
- South East (e.g. Oxford, Brighton)
- South West (e.g. Bristol Cornwall)

32. **How would you describe your ethnicity?**

- White
- Asian or Asian British
- Black, Black British, Caribbean or African
- Mixed or multiple ethnic groups
- Prefer not to say
- Other: please specify

33. **How would you describe your education background?**

- Less than GCSEs
- GCSEs
- A-levels or equivalent
- Undergraduate degree or equivalent
- Postgraduate degree or equivalent
- Prefer not to say
- Other: please specify

34. **How would you describe your employment status?**

- Student
- Employed: Full-time
- Employed: Part-time
- Unemployed
- Retired
- Prefer not to say
- Other: please specify

35. **Do you consider yourself to be a disabled person?**

- Yes
- No
- Prefer not to say

36. **Do you consider yourself to be neurodivergent? (This term refers to conditions such as ADHD, autism and dyslexia)**

- Yes
- No
- Prefer not to say

37. **Which of the following best describes your gender identity?**

- Male
- Female
- Non-binary
- Prefer not to say
- Other: please specify
