# Supplementary material for: The Impact of Heavy Menstrual Bleeding on Quality of Life: A Mixed‐Methods Survey Study
Source: BJOG. 2026 Jun 21;133(12):2170–7. doi: 10.1111/1471-0528.70290 (PMC13659367; doi:10.1111/1471-0528.70290)
Supplement: Supplementary file 5 [file BJO-133-2170-s002.docx]

## Figure S1. Social media advert for recruiting participants
